# The Book World of Medicine and Science

**Published:** 1893-07-01

**Authors:** 


### The Book World of Medicine and Science.

Homer and the Epic. By Andrew Lang. (Longmans, Green, and Co.)

Mr. Lang, summing up in this work the results of Homeric criticism from the earliest times down to the present day, offers an eloquent and (to beg the whole question) convincing proof of the substantial unity of authorship of both the "Iliad" and the "Odyssey." He starts by exposing the extremely slender premises on which Wolff's theories of the conglomerate "Iliad" are based. Wolff, it may be remembered, relied principally on the absence of any proof that Homer could write, or that he had anything to write on, or anyone to read what he had written; he inferred, consequently, that the "Iliad" was handed down by tradition, that it was added to by successive hands, and that these interpolations were tagged on to the main body of the epic by joins (juncturæ), consisting of one or more explanatory verses; that the unity of construction and artistic merit were conferred by the hand of Pisistratus, who first ordered the epics to be committed to writing, and published them in the order wherein we read them now. Subsequent scholars, principally Germans, have enlarged upon this theory. They have by common consent abandoned the Pisistratean hypothesis as not proven, but by internal evidence profess to show exactly what portions of the epics were composed by Homer, what were ancient lays adapted by him, what are more recent interpolations, and what the tags which linked the scattered fragments into a coherent whole. Mr. Lang examines in detail the arguments of these "microscope men," and they make very good reading in his hands. The extraordinary allusiveness of his style, and the energy of his irony where he finds his favourite passages "generally bedevilled," lend a piquant flavour even to the dusty bran of the German commentators. To the general spectator, taking no active part in the skirmish, but watching to see to which side the victory inclines, it will seem that Mr. Lang has the best of it all along the line. From the literary, historical, and archæological crucible his golden Homer issues almost unimpaired. The conclusion to which he works is briefly "that the hypothesis of a crowd of great harmonious poets, working for centuries at the 'Iliad,' and sinking their own fame and identity in Homer's, appears more difficult of belief than the opinion that one great poet may make occasional slips and blunders." Yet it may be observed that the difference in conception of religion and the gods which we encounter in the two epics is a difficulty which Mr. Lang does not fully meet. In a very characteristic passage he calls attention to "the very faulty human character" of the Zeus in the "Iliad:" "When we remember what this deity was —how, like Miss Austen's Mr. Bennet, he took a humorous pleasure in the absurdities of his wife and children—it really is not extraordinary he should let them disport themselves for a day before he began to fulfil a promise which he had given without enthusiasm." But in the "Odyssey" Athene is divine. It is no mere difference of attributes, as of one human character from another. It is a complete change of essence. We pass from a species of anthropomorphism to an atmosphere of something like pure faith. Could such opposite conceptions of deity appear in the work of the same man? Mr. Lang abandons the whole question of the gods as irretrievably obscure, and remarks that they hardly ever ap-

THE BOOK WORLD OF MEDICINE AND SCIENCE.—(*continued*).

pear without troubling commentators, who expect them to act like rational beings. "Their very nature is a bull and a swarm of inconsistencies." But this, again, applies almost exclusively to the "Iliad." Perhaps the student may be content to accept the compromise suggested in the introductory chapter, but not afterwards emphasised, viz., "that the Homeric epics, in spite of certain flaws and breaks, and probable insertion of alien matter, are mainly the work of one, or at the most, of *two great poets*.

### FIRST IMPRESSIONS OF BOOKS RECEIVED.

[It is requested that all books intended to be noticed in this column may be sent to the Editor, at the Office, 428, Strand, W.C., not later than Tuesday evening in each week.]

The Dublin Journal of Medical Science. June, 1893.

The surgical article is a paper by Dr. Kendal Franks, recording three cases of enterectomy and enterorrhaphy, two of which were entirely successful. The medical articles deal with simple fevers and eruptions, liable to be mistaken for infectious ones; whooping cough and measles, as registerable diseases; and the diagnostic value of the diary of reaction. The other part of the review is of its usual high merit.

The London Medical Student. By Albert Smith, author of "The Adventures of Mr. Ledbury." (London: Geo. Routledge and Sons, Limited). 1893.

This little skit was very popular when first published. It was then a caricature and not a portrait of the London medical student, and to-day bears a very faint likeness to the men found in our medical schools. To medical students the book is amusing and will not be misleading; to others it may be amusing and is very likely to be misleading, and to give an entirely false notion of the habits, mode of life, and of the work of a body of men who compare very favourably with their fellows preparing for other callings. Albert Smith was once a medical student himself at the Middlesex Hospital, and this enabled him to write with a knowledge of certain details that made his brochure attractive to medicoes.

### Books Received.

W. B. Saunders, Philadelphia.
"A Text Book of the Theory and Practice of Medicine." By William Pepper, M.D.

Religious Tract Society.
"Jessica's Mother." By Hesba Stretton.

Oxford University Press.
"Helps to the Study of the Bible."

George Routledge and Sons.

"The London Medical Student." By Albert Smith.

